# Supplementary material for: Mechanism of DNA origami folding elucidated by mesoscopic simulations
Source: Nat Commun. 2024 Apr 8;15:3015. doi: 10.1038/s41467-024-46998-y (PMC11001925; doi:10.1038/s41467-024-46998-y)
Supplement: Supplementary file 1 — Supplementary Information [file 41467_2024_46998_MOESM1_ESM.pdf]

Supplementary Information

Mechanism of DNA origami folding elucidated by mesoscopic simulations

Marcello DeLuca[1], Daniel Duke[1], Tao Ye[2,3], Michael Poirier[4], Yonggang Ke[5], Carlos Castro[6], Gaurav Arya[1]*

1. *Thomas Lord Department of Mechanical Engineering and Materials Science, Duke University, Durham, NC 27705, USA*
2. *Department of Chemistry & Biochemistry, University of California, Merced, CA 95343, USA*
3. *Department of Materials and Biomaterials Science & Engineering, University of California, Merced, CA 95343, USA*
4. *Department of Physics, The Ohio State University, Columbus, OH 43210, USA*
5. *Department of Biomedical Engineering, Georgia Institute of Technology and Emory University, Atlanta, GA 30322, USA*
6. *Department of Mechanical and Aerospace Engineering, The Ohio State University, Columbus, OH 43210, USA*

* Corresponding author
Email: gaurav.arya@duke.edu

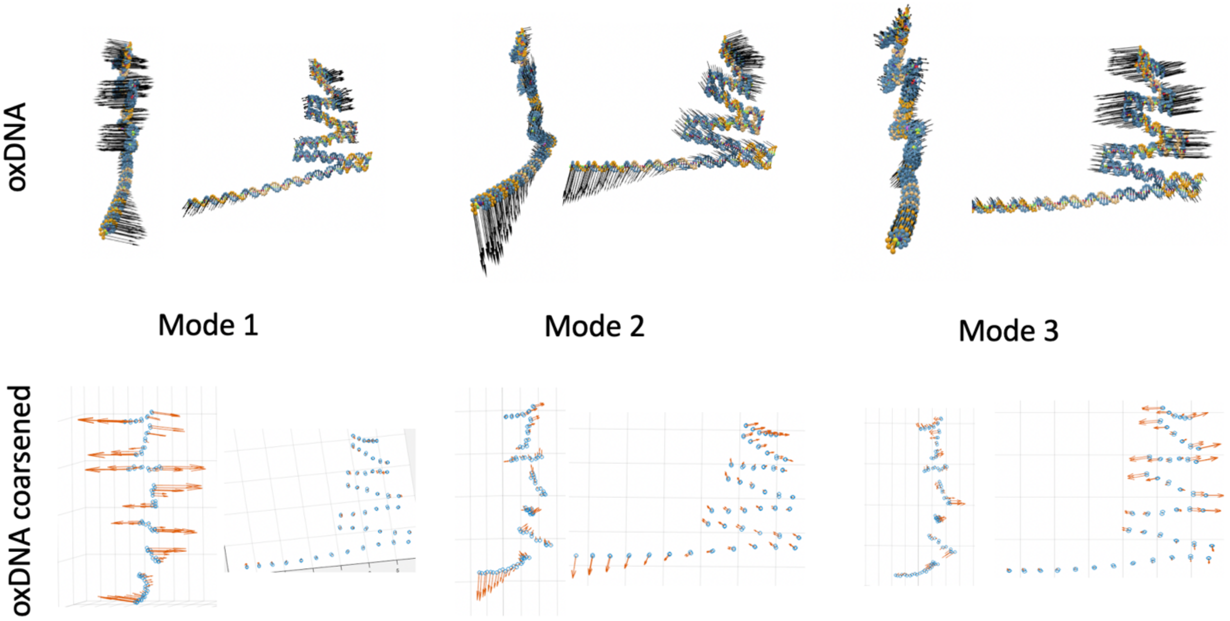

Supplementary Figure 1: First three principal components of sheet structure extracted from oxDNA simulations using single-nucleotide trajectory resolution compared to coarsened trajectory extracted from the same simulation. Results show good agreement, indicating that a model coarsened to 8 nucleotides per bead may be sufficient to capture the dynamics of DNA origami structures.

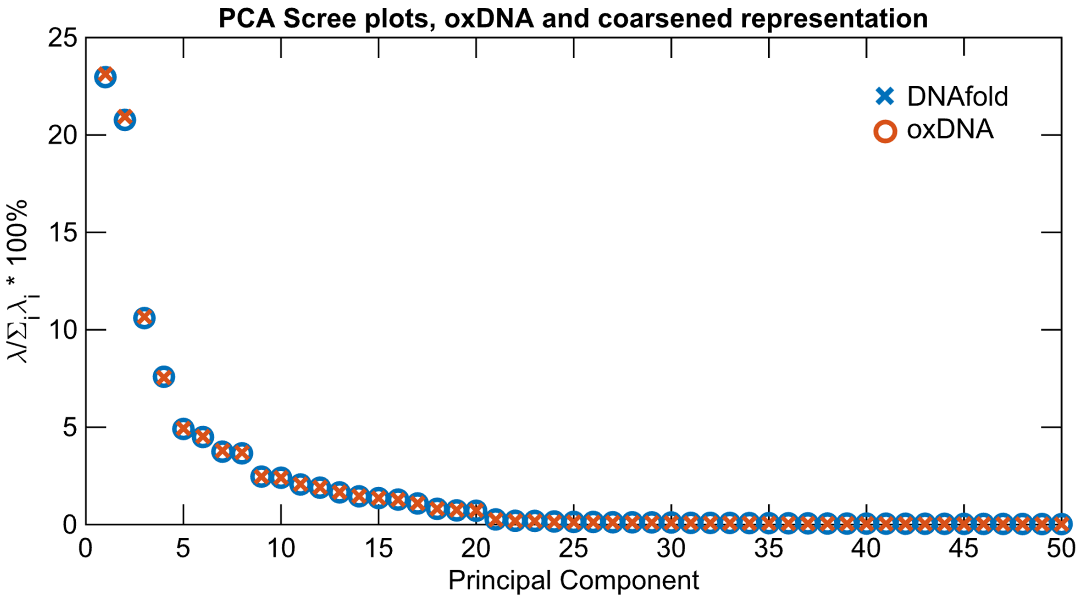

Supplementary Figure 2: Scree plots of first 50 principal components from PCA, oxDNA and mapped to the mesoscopic representation used in the DNAfold software.

Supplementary Note 1: Viability of using Brownian dynamics for this model.

The mass of each bead, which corresponds to 8 nucleotides, is approximately $2.64\,\mathrm{kDa}$ or $4.384 \times 10^{-24}\ \mathrm{kg}$. The Stokes friction coefficient of a spherical bead in a fluid is $\gamma = 6\pi\eta R$, where $R = 2.7\ \mathrm{nm}$ is the bead's radius and η is the dynamic viscosity of the surrounding fluid (water), or approximately $2.545 \times 10^{-11}\ \mathrm{kg}/s$ at room temperature. It is typically considered appropriate to assume overdamped Langevin (Brownian) dynamics if the characteristic relaxation time is smaller than the timestep of motion, $m/\gamma < \Delta t_{\mathrm{step}}$. In our case, $m/\gamma \approx 1.723 \times 10^{-13}\ \mathrm{s}$, which is approximately nine times smaller than our timestep $\Delta t_{\mathrm{step}} = 15 \times 10^{-12}\ \mathrm{s}$, and therefore it is acceptable to use Brownian dynamics for this model to more efficiently sample system dynamics.

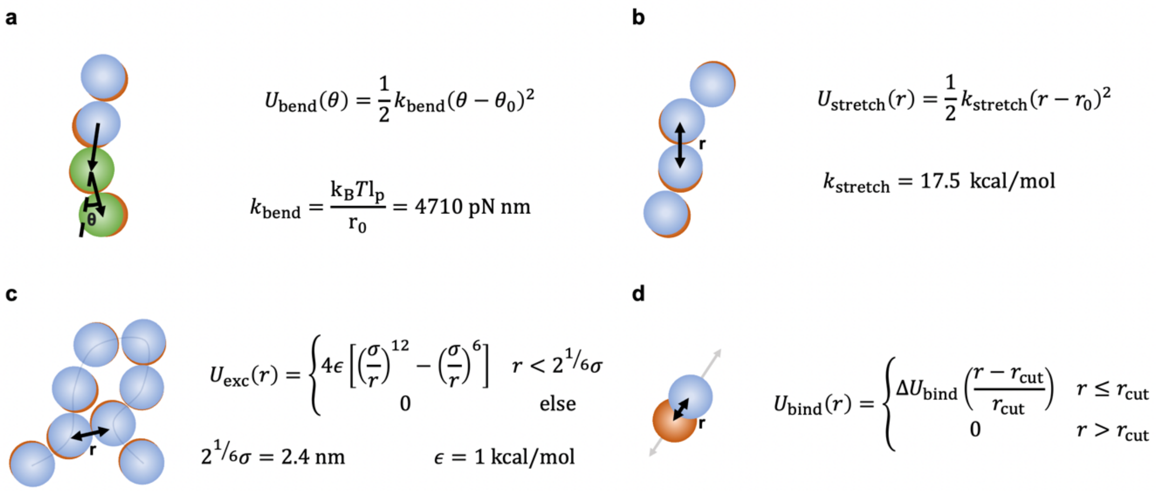

Supplementary Figure 3: Schematic of the four main potentials used in our mesoscopic model, including (a) bending potential, (b) harmonic backbone potential, (c) WCA excluded volume potential, and (d) hybridization potential.

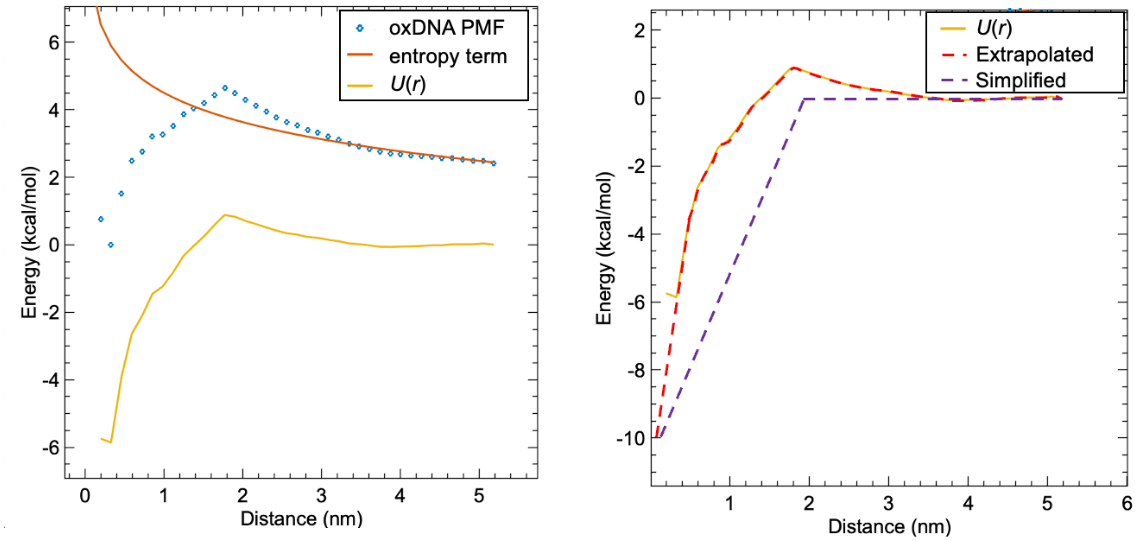

Supplementary Figure 4: The hybridization potential was derived using oxDNA-based umbrella sampling simulations of two complementary 8-nt sequences (using the sequence-independent model of oxDNA) being dehybridized. Distance denotes separation between centers of mass (COM) of the two 8-nt sequences and "Energy" denotes energy, PMF, or entropy. We then extracted the entropic term for separation, fitted it to the tail of the PMF curve, and subtracted this from the PMF to obtain hybridization energy as a function of COM separation.

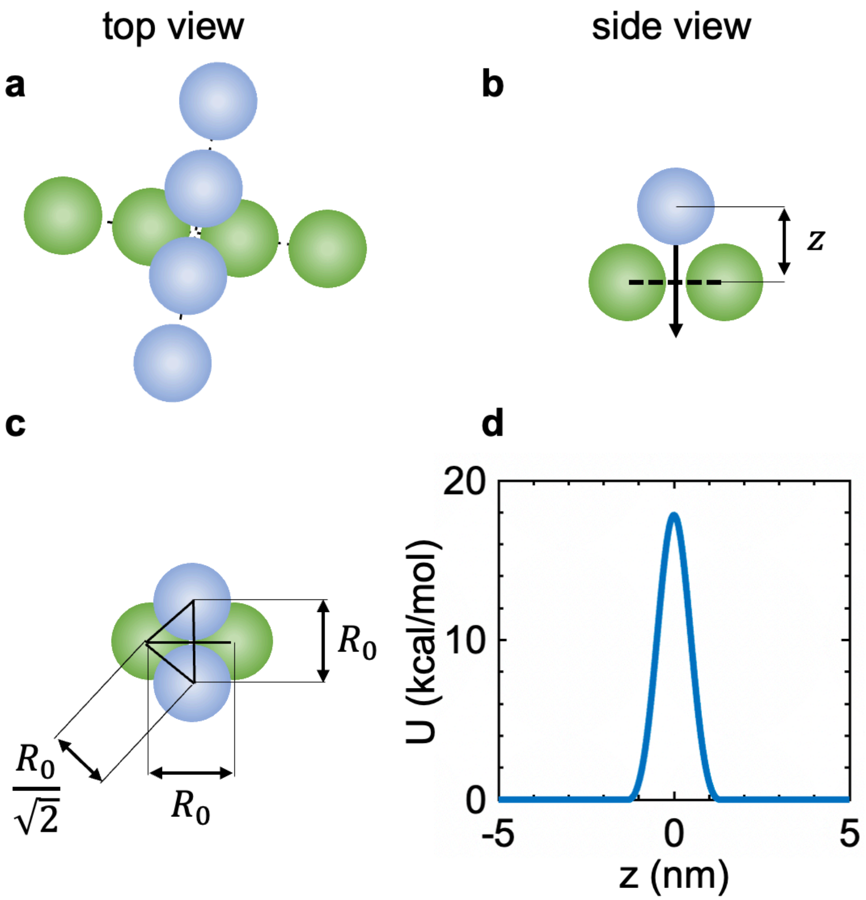

Supplementary Figure 5: (a-b) One primary function of the excluded volume potential is to prevent strand cross-through. The worst-case configuration for cross-through is one where a pair of consecutive particles approaches another pair of consecutive particles at a 90-degree angle forming a diamond pattern; this minimizes the excluded volume penalty to cross-through. (c) Geometry showing two beads located at $R_0/\sqrt{2}$ from two other beads (at height *z*=0) is the location of maximum potential and represents the minimum barrier to cross-through. (d) cross through potential arising from excluded volume interactions as a function of *z*, the height of one strand above the other, in this case depicted by blue above green.

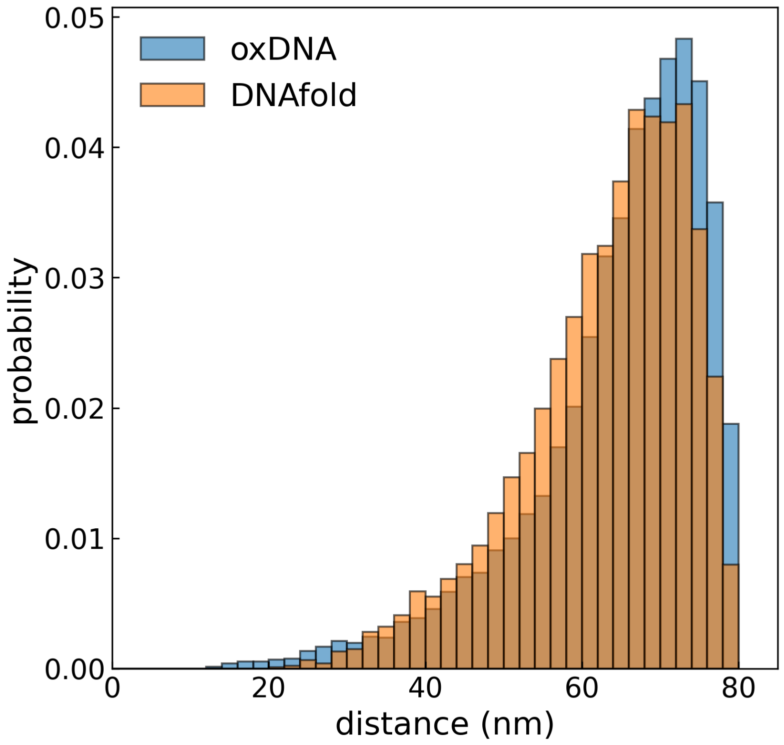

Supplementary Figure 6: End-to-end distance comparison between mesoscopic (DNAfold) and oxDNA models for a 256-base pair section of dsDNA.

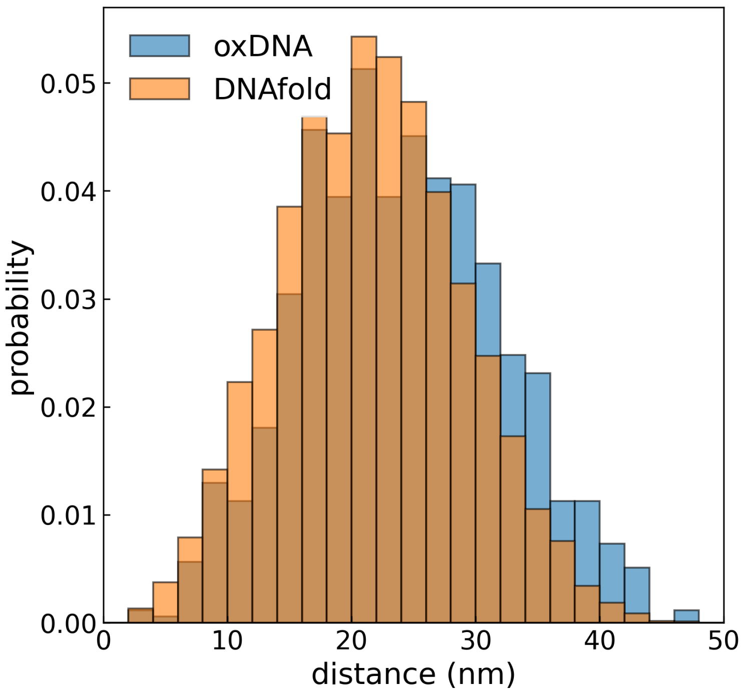

Supplementary Figure 7: End-to-end distance distribution comparison between DNAfold and oxDNA for a 256-nucleotide ssDNA section. Since this version of the model is sequence independent, we used polythymine as a reference.

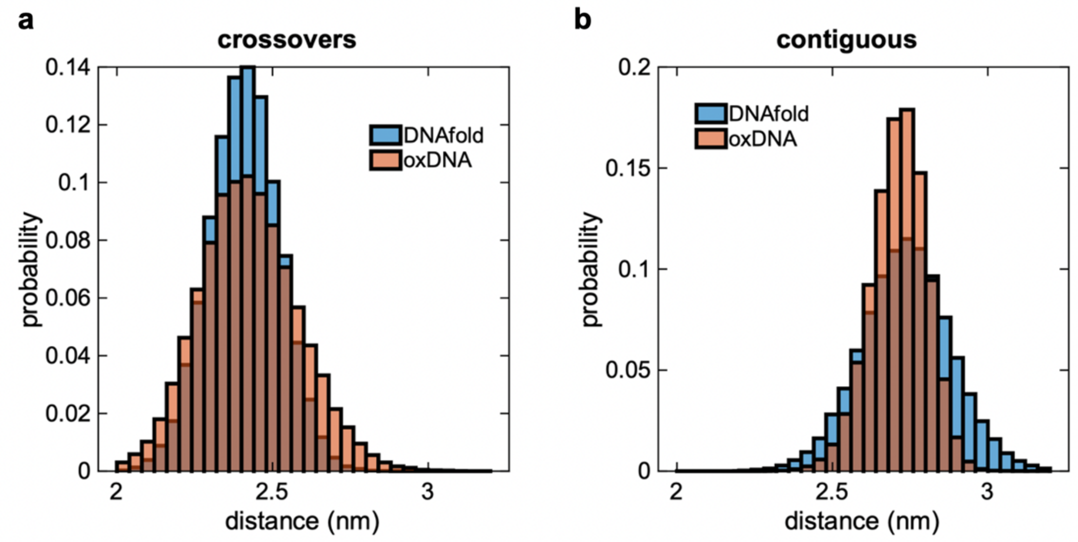

Supplementary Figure 8: Comparison of backbone length distributions from oxDNA to DNAfold for a fully assembled 768-nucleotide sheet structure. (a) distribution of separation distances between crossover beads. (b) distribution of separation distances between contiguous beads.

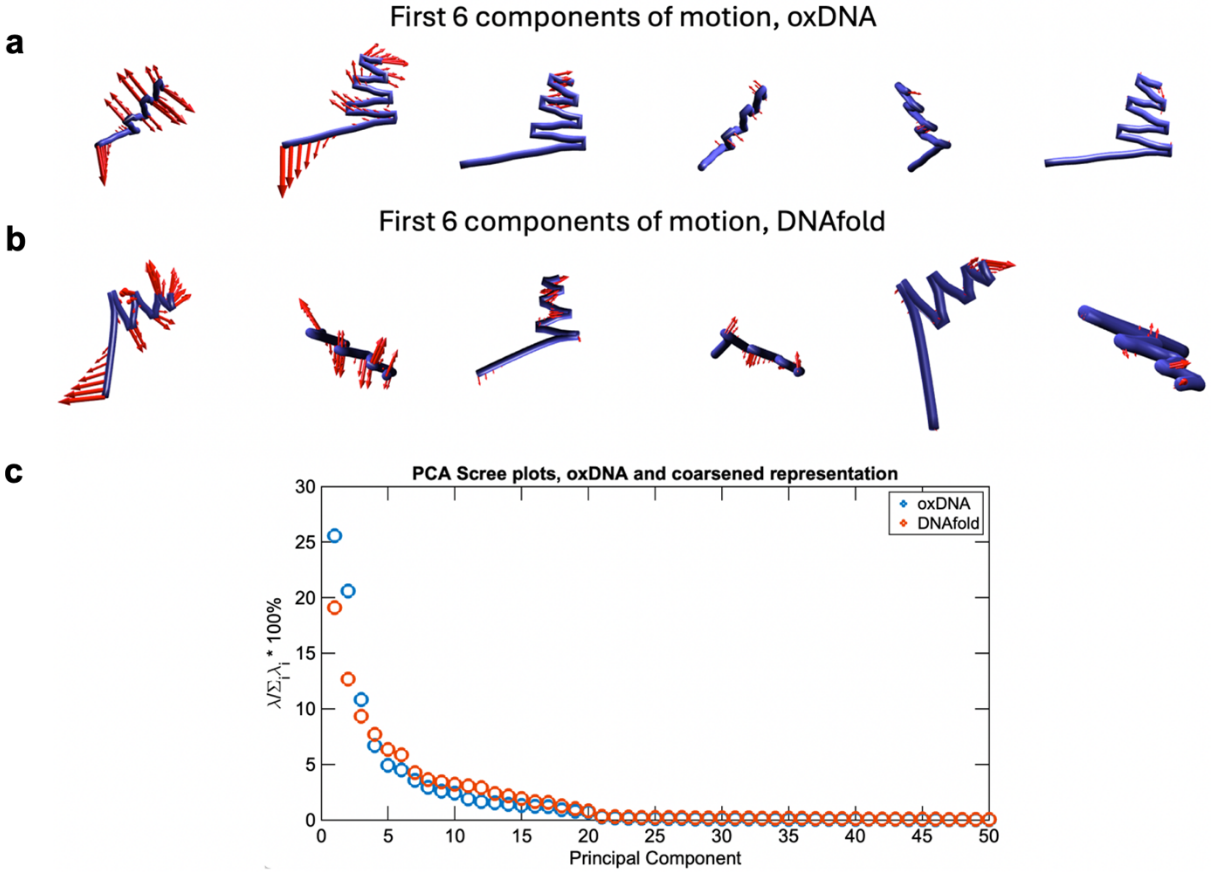

Supplementary Figure 9: Comparison of principal components and scree plots for oxDNA and DNAfold. (a) first 6 PCs for oxDNA sheet structure. (b) first 6 PCs for DNAfold sheet structure. (c) scree plots for both models mapped to same representation.

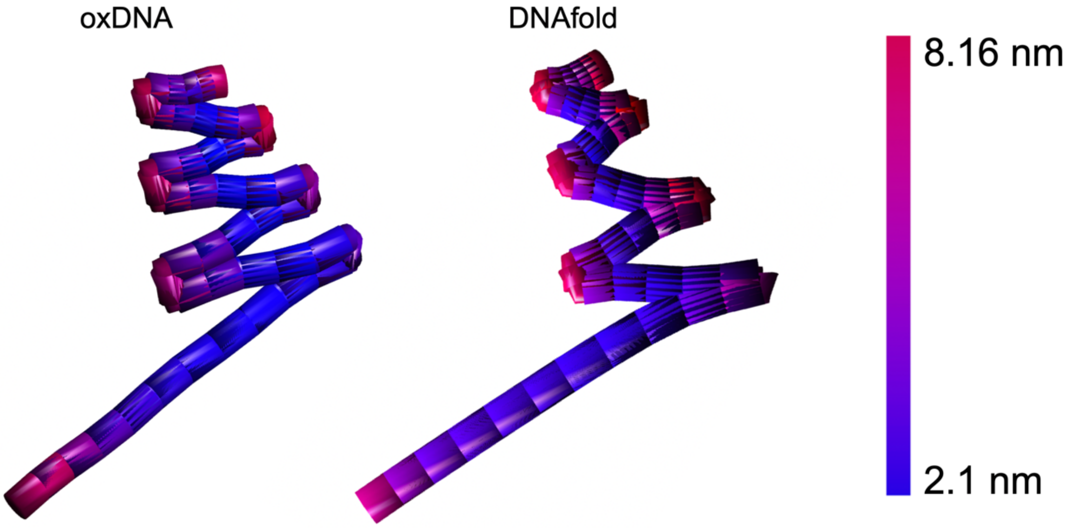

Supplementary Figure 10: RMSF comparison between oxDNA and DNAfold.

Standard 4HB design

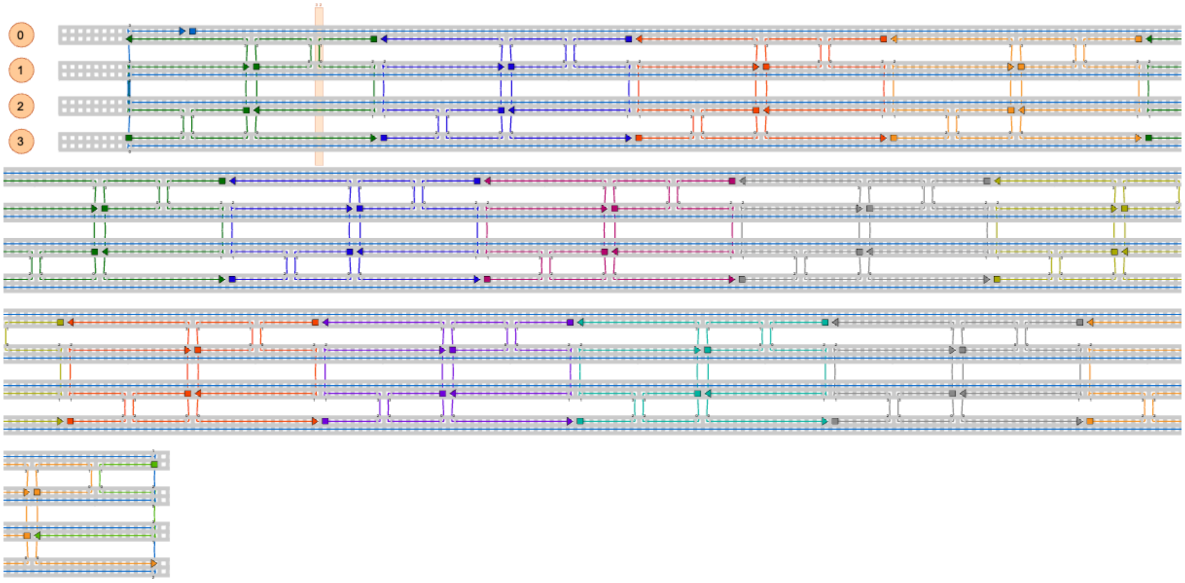

Supplementary Figure 11: caDNAno representation of standard four helix bundle (4HB) design.

4HB, Seam Scaffold

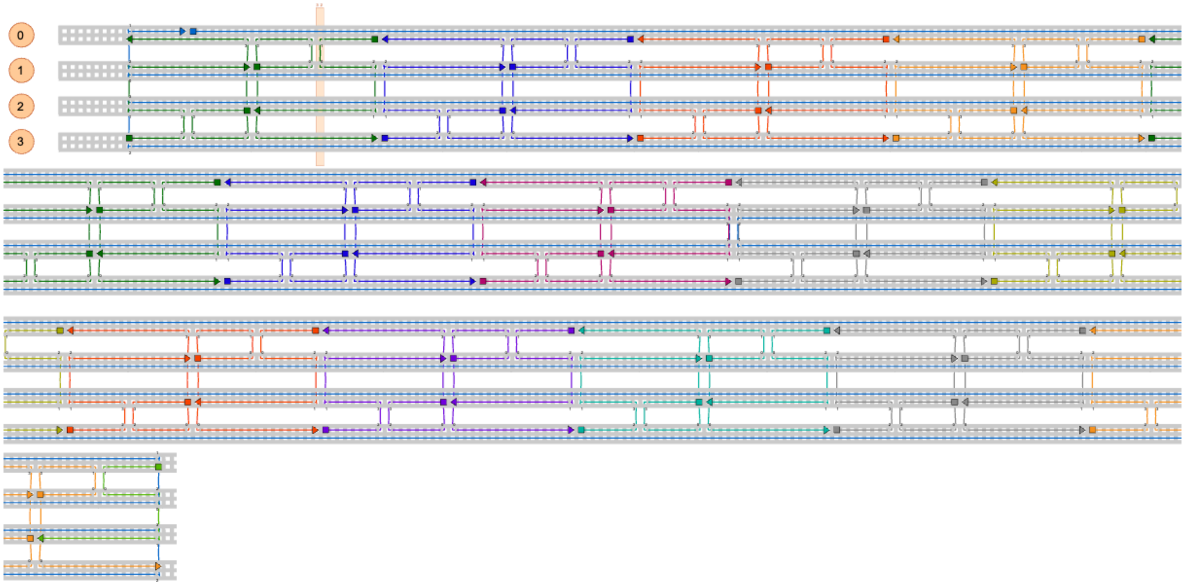

Supplementary Figure 12: caDNAno representation of 4HB design with seamed scaffold.

4HB, Winding Scaffold

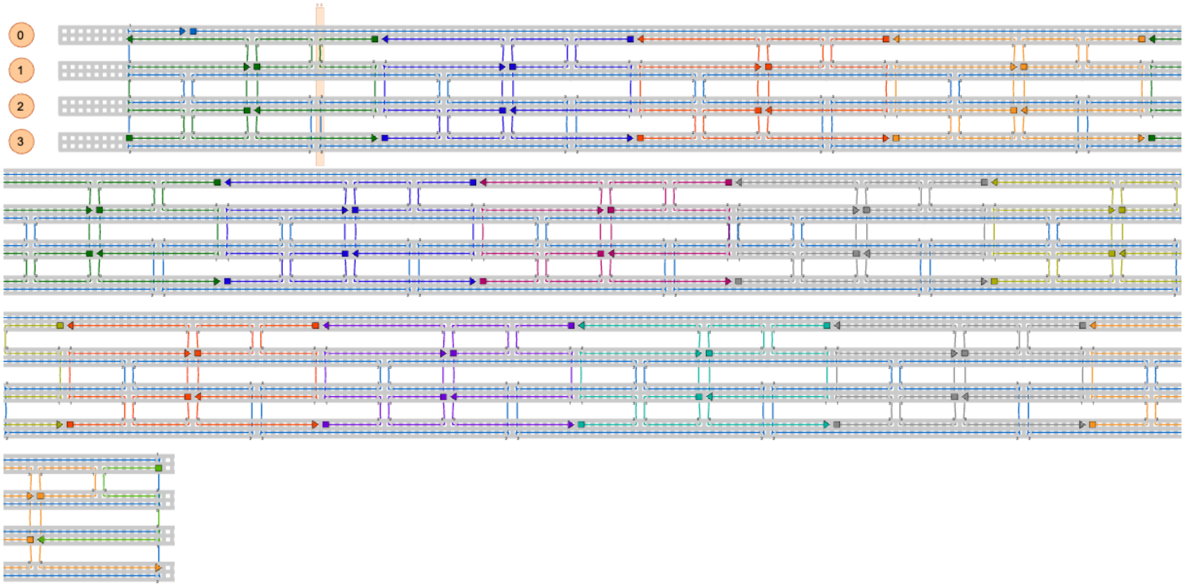

Supplementary Figure 13: caDNAno representation of 4HB design with winding scaffold.

4HB, Long Center Staples

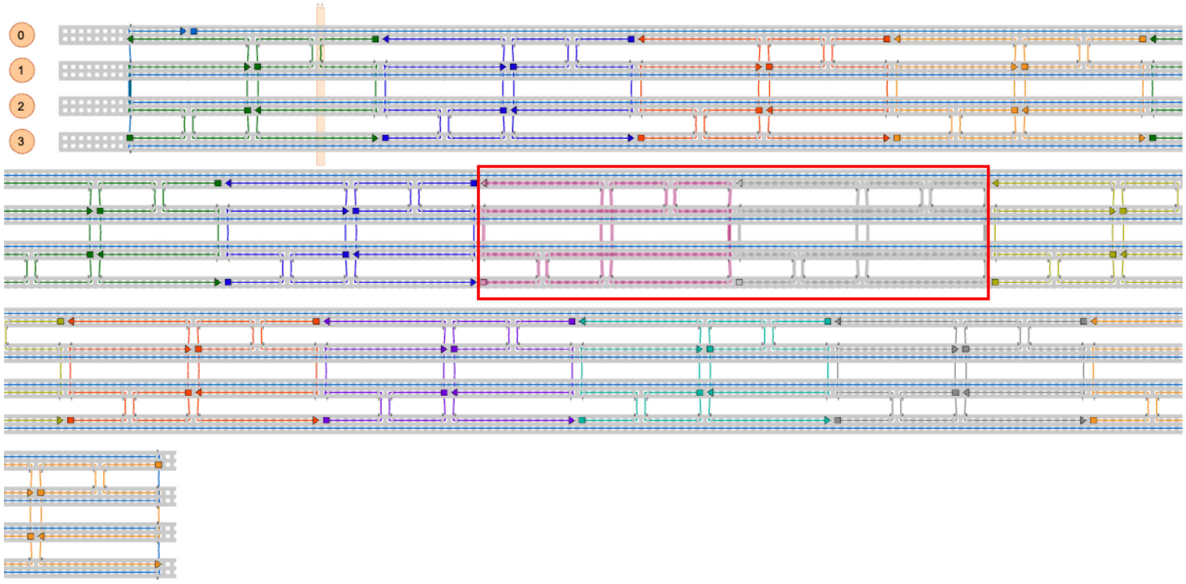

Supplementary Figure 14: 4HB with long center staples, shown in bold pink and grey and boxed in red.

4HB, Long End Staples

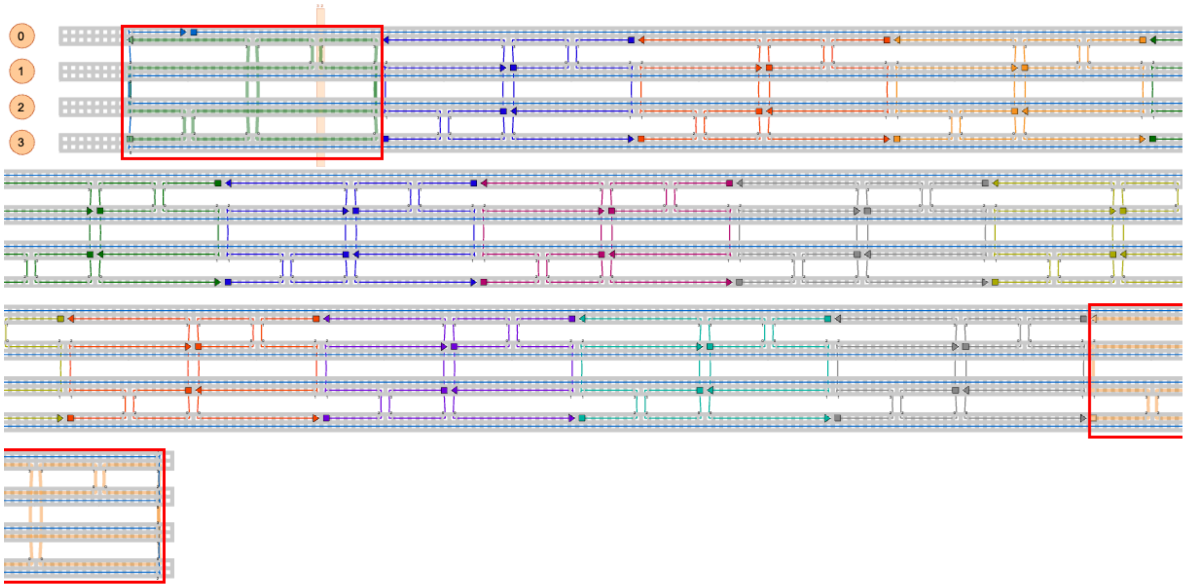

Supplementary Figure 15: 4HB with long end staples, shown in bold green and orange and boxed in red.

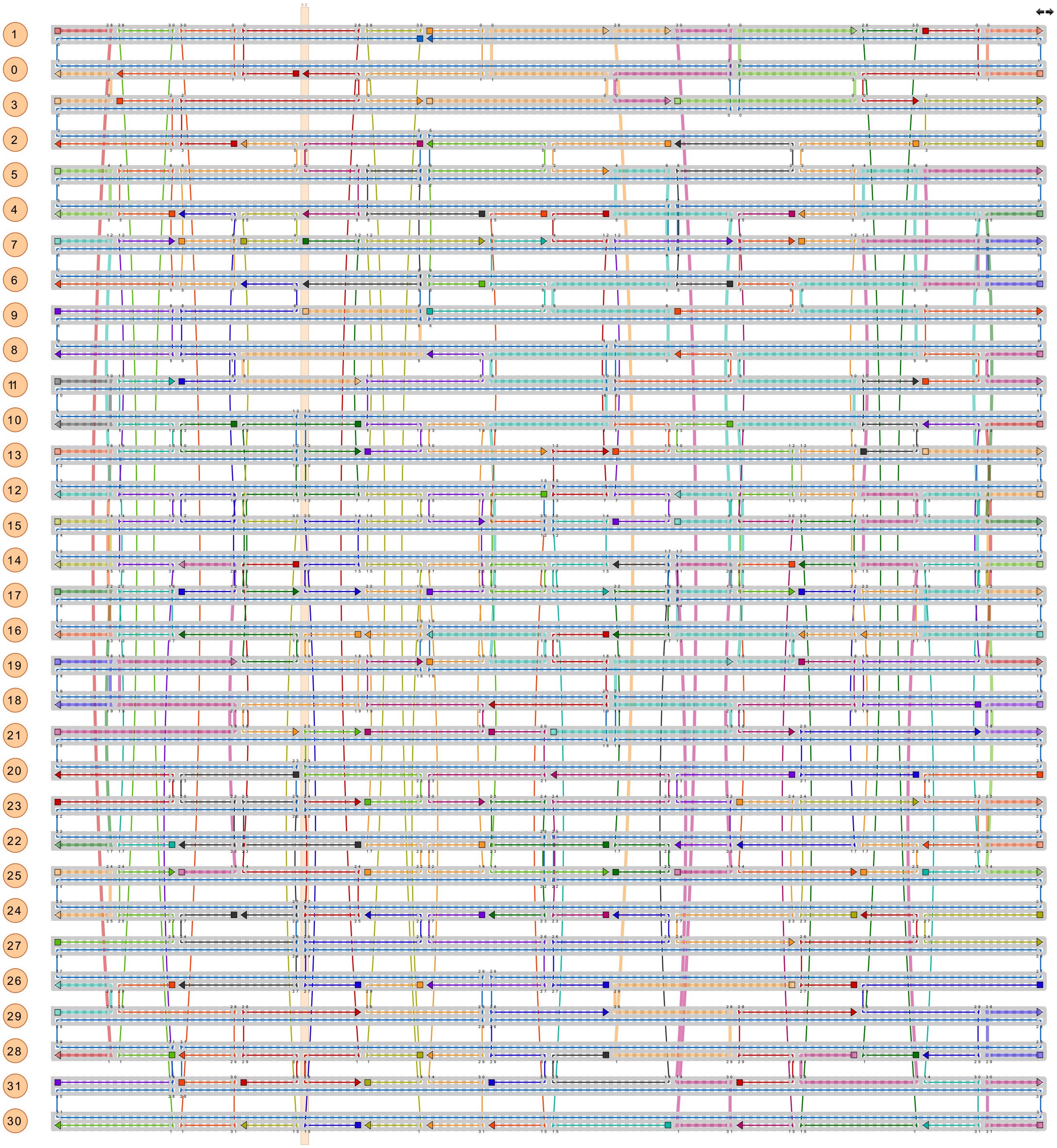

Supplementary Figure 16: caDNAno representation of 32 Helix Bundle design

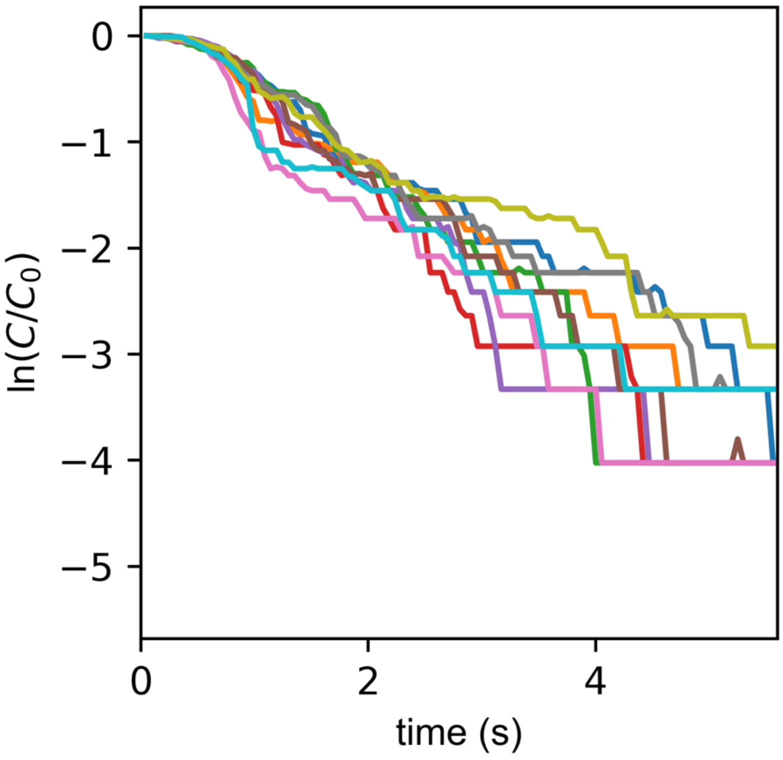

Supplementary Figure 17: individual kinetic traces for 4HB with straight scaffold routing and hybridization strength of 10 kcal/mol per bead.

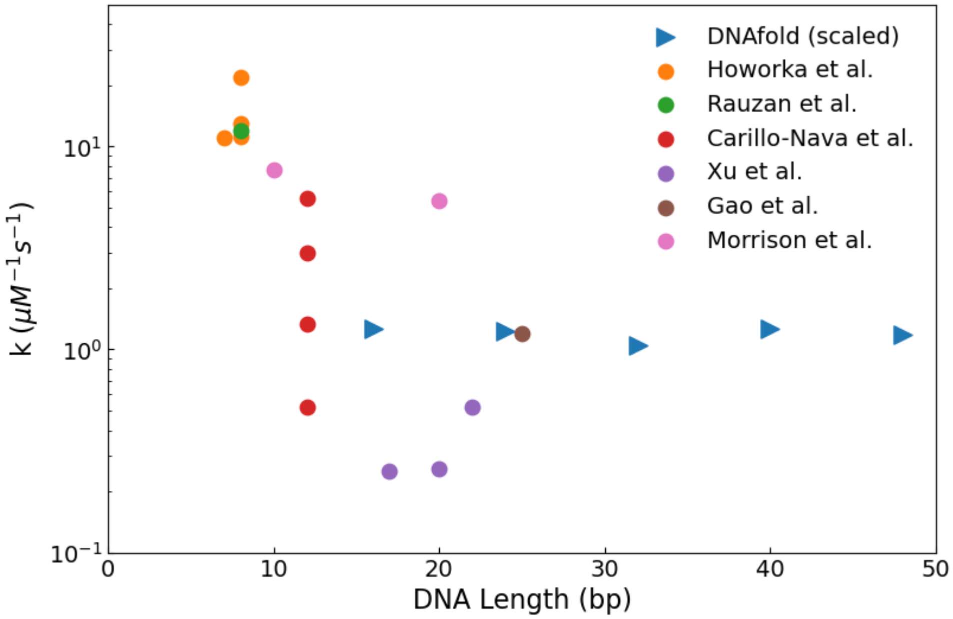

Supplementary Figure 18: Comparison of simulated (using our mesoscopic DNAfold model) and experimental hybridization rate constants[1-6] for dsDNA at various strand lengths. The simulated values include a 5200x time scaling. Multiple experimental data points from the same study at the same DNA length indicates strands with different sequences. According to our DNAfold simulations, the rate constant does not significantly change with DNA length within the tested range. This agrees with experimental data,[7] which indicates that the hybridization rate constant is independent of strand length for dsDNA with more than 8 base pairs.

Supplementary Table 1: Model Parameters

Potential	Type of DNA	Form	Parameter 1 (kcal/mol, kcal/mol/nm^2)	Parameter 2 (nm, radians)
Backbone	dsDNA and ssDNA	$U_{\text{stretch}} = \frac{1}{2} k_{\text{stretch}} (r - r_0)^2$	$k_{\text{stretch}} = 21.8$	$r_0 = 2.725$
Backbone	Crossovers	$U_{\text{stretch}} = \frac{1}{2} k_{\text{stretch}} (r - r_0)^2$	$k_{\text{stretch}} = 21.8$	$r_0 = 2.4$
Bending	ssDNA	0	0	0
Bending	dsDNA scaffold	$U_{\text{bend}} = \frac{1}{2} k_{\text{bend}} (\theta - \theta_0)^2$	$k_{\text{bend}} = 10.91$	$\theta_0 = 0$
Bending	Scaffold Crossover	$U_{\text{bend}} = \frac{1}{2} k_{\text{bend}} (\theta - \theta_0)^2$	$k_{\text{bend}} = 10.91$	$\theta_0 = \pi/2$
Excluded Volume	Non-complementary beads of same type (scaffold-scaffold or staple-staple)	$U_{\text{WCA}} = \begin{cases} 4\varepsilon \left[\left(\frac{\sigma}{r}\right)^{12} - \left(\frac{\sigma}{r}\right)^{6} \right] + \varepsilon & r \le 2^{1/6}\sigma \\ 0 & r > 2^{1/6}\sigma \end{cases}$	$\varepsilon = 1$	$\sigma = 2.07356$
Crossover Stabilization	Scaffold crossover	$U(r)_{\text{cs}} = \frac{1}{2} k_{\text{stretch}} (r_{i,i+2} - r_0)^2$	$k_{\text{stretch}} = 87.4$	$r_0 = 3.6312$
Hybridization	Complementary pairs	$U_{\text{bind}}(r) = \begin{cases} \Delta U_{\text{bind}} \left(\frac{r - r_{\text{cut}}}{r_{\text{cut}}} \right) & r \le r_{\text{cut}} \\ 0 & r > r_{\text{cut}} \end{cases}$	$\Delta U_{\text{bind}} = -10$	$r_{\text{cut}} = 2$

Supplementary References

1 Carrillo-Nava, E., Mejía-Radillo, Y. & Hinz, H.-J. r. Dodecamer DNA duplex formation is characterized by second-order kinetics, positive activation energies, and a dependence on sequence and Mg2+ ion concentration. *Biochemistry* **47**, 13153-13157 (2008).

2 Gao, Y., Wolf, L. K. & Georgiadis, R. M. Secondary structure effects on DNA hybridization kinetics: a solution versus surface comparison. *Nucleic Acids Res.* **34**, 3370-3377 (2006).

3 Howorka, S., Movileanu, L., Braha, O. & Bayley, H. Kinetics of duplex formation for individual DNA strands within a single protein nanopore. *Proc. Natl. Acad. Sci. USA* **98**, 12996-13001 (2001).

4 Morrison, L. E. & Stols, L. M. Sensitive fluorescence-based thermodynamic and kinetic measurements of DNA hybridization in solution. *Biochemistry* **32**, 3095-3104 (1993).

5 Rauzan, B. *et al.* Kinetics and thermodynamics of DNA, RNA, and hybrid duplex formation. *Biochemistry* **52**, 765-772 (2013).

6 Xu, S. *et al.* Real-time reliable determination of binding kinetics of DNA hybridization using a multi-channel graphene biosensor. *Nat. Commun.* **8**, 14902 (2017).

7 Zhang, D. Y. & Winfree, E. Control of DNA strand displacement kinetics using toehold exchange. *J. Am. Chem. Soc.* **131**, 17303-17314 (2009).
