# Supplementary material for: Mechanism of DNA origami folding elucidated by mesoscopic simulations
Source: Nat Commun. 2024 Apr 8;15:3015. doi: 10.1038/s41467-024-46998-y (PMC11001925; doi:10.1038/s41467-024-46998-y)
Supplement: Supplementary file 3 — Description of Additional Supplementary Files [file 41467_2024_46998_MOESM3_ESM.pdf]

Description of Additional Supplementary Files

Supplementary Movie 1: Straight-routed 4-helix bundle with 6 kcal/mol hybridization strength.

Supplementary Movie 2: Straight-routed 4-helix bundle with 8 kcal/mol hybridization strength.

Supplementary Movie 3: Straight-routed 4-helix bundle with 10 kcal/mol hybridization strength.

Supplementary Movie 4: Straight-routed 4-helix bundle with 12 kcal/mol hybridization strength.

Supplementary Movie 5: Seam-routed 4-helix bundle with 10 kcal/mol hybridization strength.

Supplementary Movie 6: Winding-routed 4-helix bundle with 10 kcal/mol hybridization strength.

Supplementary Movie 7: Straight-routed 4-helix bundle with 10 kcal/mol hybridization strength and elongated center staples exhibiting sudden collapse.

Supplementary Movie 8: Straight-routed 4-helix bundle with 10 kcal/mol hybridization strength and elongated center staples exhibiting slower zipping.

Supplementary Movie 9: Straight-routed 4-helix bundle with 10 kcal/mol hybridization strength and elongated end staples.

Supplementary Movie 10: 32-helix bundle with 10 kcal/mol hybridization strength exhibiting poor folding.

Supplementary Movie 11: 32-helix bundle with 10 kcal/mol hybridization strength exhibiting ideal folding.
